# Severe Acute Respiratory Syndrome Coronavirus 2 Serosurveillance in a Patient Population Reveals Differences in Virus Exposure and Antibody-Mediated Immunity According to Host Demography and Healthcare Setting

**DOI:** 10.1093/infdis/jiaa788

**Published:** 2020-12-26

**Authors:** Ellen C Hughes, Julien A R Amat, Joanne Haney, Yasmin A Parr, Nicola Logan, Norah Palmateer, Sema Nickbakhsh, Antonia Ho, Peter Cherepanov, Annachiara Rosa, Andrew McAuley, Alice Broos, Imogen Herbert, Ursula Arthur, Agnieszka M Szemiel, Chloe Roustan, Elizabeth Dickson, Rory N Gunson, Mafalda Viana, Brian J Willett, Pablo R Murcia

**Affiliations:** 1 MRC–University of Glasgow Centre for Virus Research, Institute of Infection, Immunity and Inflammation, College of Medical, Veterinary and Life Sciences, University of Glasgow, Glasgow, United Kingdom; 2 Institute of Biodiversity, Animal Health and Comparative Medicine, College of Medical, Veterinary and Life Sciences, University of Glasgow, Glasgow, United Kingdom; 3 School of Veterinary Medicine, College of Medical, Veterinary and Life Sciences, University of Glasgow, Glasgow, United Kingdom; 4 Public Health Scotland (Health Protection Scotland), Glasgow, United Kingdom; 5 School of Health $ Life Sciences, Glasgow Caledonian University, Glasgow, United Kingdom; 6 Chromatin Structure and Mobile DNA Laboratory, The Francis Crick Institute, London, United Kingdom; 7 Department of Medicine, Imperial College London, St Mary’s Campus, London, UK; 8 West of Scotland Specialist Virology Centre, NHS Greater Glasgow and Clyde, Glasgow, United Kingdom

**Keywords:** SARS-CoV-2, COVID-19, virus exposure, serology, virus neutralization, modelling, risk factors, seroprevalence

## Abstract

Identifying drivers of severe acute respiratory syndrome coronavirus 2 (SARS-CoV-2) exposure and quantifying population immunity is crucial to prepare for future epidemics. We performed a serial cross-sectional serosurvey throughout the first pandemic wave among patients from the largest health board in Scotland. Screening of 7480 patient serum samples showed a weekly seroprevalence ranging from 0.10% to 8.23% in primary and 0.21% to 17.44% in secondary care, respectively. Neutralization assays showed that highly neutralizing antibodies developed in about half of individuals who tested positive with enzyme-linked immunosorbent assay, mainly among secondary care patients. We estimated the individual probability of SARS-CoV-2 exposure and quantified associated risk factors. We show that secondary care patients, male patients, and 45–64-year-olds exhibit a higher probability of being seropositive. The identification of risk factors and the differences in virus neutralization activity between patient populations provided insights into the patterns of virus exposure during the first pandemic wave and shed light on what to expect in future waves.

## Teaser

A serial cross-sectional serosurvey among primary and secondary care patients in Glasgow, Scotland, shows low seroprevalence during the first wave of the coronavirus disease 2019 pandemic and identifies age, sex and care type as factors associated with increased probability of infection.

Severe acute respiratory syndrome coronavirus 2 (SARS-CoV-2) was first reported in China in December 2019 and spread rapidly across multiple countries. The first coronavirus disease 2019 (COVID-19) case in Scotland was confirmed on 28 February 2020, the country entered lockdown on 23 March, and restrictions were eased on 28 May [1]. Serological surveys are instrumental in determining infection rates at the population scale [2]. Assays based on the detection of anti-SARS-CoV-2 immunoglobulin (Ig) G antibodies, which are typically detectable 7–21 days after infection [3], can identify past viral exposure even in asymptomatic individuals. In-house assays commonly use an indirect enzyme-linked immunosorbent assay (ELISA) format, with recombinant S protein, S1 subunit of the S protein, or the receptor-binding domain (RBD) used as antigens. Virus neutralization assays provide insights into the effectiveness of the humoral immune response. Neutralization titers obtained with pseudotype-based tests are similar to those obtained with live virus [4], and 2 pseudotype-based methods are commonly used: human immunodeficiency virus (HIV)–based and vesicular stomatitis virus–based pseudotypes. Both methods produce similar results [5].

Models that link patient information (eg, age, sex, and time of sampling) with exposure and immunity enable the identification of factors associated with SARS-CoV-2 infection [6]. NHS Greater Glasgow and Clyde (NHSGGC) is the largest health board in Scotland and reported the most COVID-19 cases (n = 3876) and deaths (n = 1280) in the country between 1 March and 24 May [7]. We performed a serial cross-sectional study among primary and secondary care patients in NHSGGC to estimate levels of exposure to SARS-CoV-2 since the introduction of the virus in Scotland and up to calendar week 21 (starting on 18 May 2020). Using a bayesian framework, we combined serological and patient information to estimate an individual’s probability of testing positive for SARS-CoV-2 across various age groups, time and healthcare settings. We also performed neutralization assays to estimate the fraction of exposed individuals who developed an effective antibody response. Finally, we combined serological data with publicly available information on deaths to estimate the case-fatality ratio.

## METHODS

### Serum Samples

Ethical approval was provided by NHSGGC Biorepository (application 550). Random residual biochemistry serum samples (n = 7480) from primary (general practices) and secondary (hospitals) healthcare settings were collected by the NHSGGC Biorepository between 16 March and 24 May 2020. Associated metadata included date of collection, patient sex and age, partial postal code of the patient, and sample origin (primary or secondary care). All serum samples were inactivated at 56ºC for 30 minutes before being tested.

### ELISA Testing

S1 and RBD antigens were prepared as described elsewhere [8]. The SARS-CoV-2 RBD and S1 constructs, spanning SARS-CoV-2 S (UniProt ID P0DTC2) residues 319–541 (RVQPT…KCVNF) and 1–530 (MFVFL…GPKKS), respectively, were produced with C-terminal twin Strep tags. Proteins were produced by transient expression in Expi293F cells grown in FreeStyle-293 medium (Thermo Fisher Scientific). Proteins were harvested at 2 time points, 3–4 and 6–8 days after transfection. Twin Strep-tagged proteins were captured on Streptactin XT (IBA LifeSciences) and purified by size exclusion chromatography through Superdex 200 (GE Healthcare). Purified SARS-CoV-2 antigens, concentrated to 1–5 mg/mL by ultrafiltration were aliquoted and snap-frozen in liquid nitrogen before storage at −80ºC.

Assays to detect IgG antibodies against recombinant S1 and RBD antigens of SARS-CoV-2 were performed as described elsewhere [9]. First, 96-well plates (Immulon 2HB, Fisher Scientific) were coated overnight with S1 or RBD antigen (50 ng per well). After being washed 3 times with phosphate-buffered saline (PBS)/0.05% Tween 20 (all subsequent wash steps followed the same protocol), serum samples were diluted 1:100 in PBS/0.05% Tween 20 (vol/vol) supplemented with 10% (vol/vol) casein (Vector Laboratories; 2BScientific) and incubated for 1 hour at room temperature before a second wash. Anti-human IgG horseradish peroxidase–conjugated secondary antibody (Bethyl Laboratories) diluted 1:3000 in PBS/0.05% Tween 20/casein was then added and incubated for 1 hour before a third wash. Next, 3,3′,5,5′-tetramethylbenzidine (Sigma-Aldrich/Merck) was added and incubated for 10 minutes in the dark.

The reaction was stopped by adding an equal volume of 1-mol/L sulfuric acid. Absorbance was read immediately at 450 nm on a Labsystems Multiskan Ascent plate reader. Duplicates of pooled known-positive and known-negative controls were included on each plate. Raw absorbance values were corrected using the following equation: (sample absorbance − negative control mean)/negative control mean. This value was used for downstream analysis. The cutoff between positive and negative values was selected using receiver operating characteristic (ROC) analysis undertaken with the corrected absorbance values of positive and negative control samples tested on the assay. A total of 320 serum samples collected before December 2019, obtained from the National Institute for Biological Standards and Control and the Scottish National Blood Transfusion Service, were used as negative controls.

Positive controls were defined as samples from patients with a positive reverse-transcription polymerase chain reaction result, or those who had recent clinical symptoms consistent with COVID-19 and whose serum sample tested positive on all other serological platforms (EUROIMMUN-Anti-SARS-CoV-2 ELISA [IgG], Abbott Architect SARS-CoV-2 IgG, or DiaSorin LIAISON SARS-CoV-2 S1/S2 IgG). A total of 128 samples were used as positive controls. Cutoff values for individual antigens were chosen to optimize for the specificity of each individual test, while maintaining a sensitivity >90%. All samples were tested against both S1 and RBD antigens, and separate ROC analyses were undertaken for each antigen. ROC analyses were performed using GraphPad Prism software (v9.0.0) (GraphPad) (Supplementary Figure 1). Final sensitivity and specificity values, and 95% confidence intervals (CIs), were calculated by applying the individual cutoff values for S1 and RBD, derived from the ROC analysis, to the control samples in parallel (ie, if a sample tested positive for either or both antigens, it was considered positive). The resulting numbers of true-positives and true-negatives, and false-positives and false-negatives, were then used to calculate the final sensitivity and specificity of the combined assays.

### Neutralization Assays

HEK293, HEK293T, and 293–angiotensin-converting enzyme 2 (ACE2) cells were maintained in Dulbecco’s modified Eagle’s medium (DMEM) supplemented with 10% fetal bovine serum, 2-mmol/L L-glutamine, 100-µg/mL streptomycin and 100-IU/mL penicillin. HEK293T cells were transfected with the SARS-CoV-2 S (corresponding to Wuhan-Hu-1 strain; GenBank MN908947) gene expression vector pCDNA6-S (from N. Temperton, University of Kent), together with pNL4-3-Luc-E^−^R^−^luc [10] using polyethylenimine (Polysciences). HIV (SARS-CoV-2)–containing supernatants were harvested 48 hours after transfection, aliquoted and frozen at −80^º^C before use. 293-ACE2 target cells were generated by stable transduction of HEK293 cells with pSCRPSY-human ACE2 (hACE2). Selected 293-ACE2 cells were maintained in complete DMEM supplemented with 2-µg/mL puromycin.

Neutralizing antibodies were measured using a fixed dilution screening. Duplicate serum samples were diluted 1:50 in complete DMEM and incubated for 1 hour with an equal volume of HIV (SARS-CoV-2) pseudotypes. The serum-virus mix was plated onto 293-ACE2 cells in 96-well white cell culture plates. After 48–72 hours, luciferase activity was quantified by adding Steadylite Plus chemiluminescence substrate (Perkin Elmer) and analyzed on a Perkin Elmer EnSight multimode plate reader (Perkin Elmer). Serum samples were considered to have high neutralizing activity if at a 1:50 dilution they reduced infection by HIV (SARS-CoV-2) pseudotypes by ≥90% [11].

### COVID-19 Data

The number of laboratory-confirmed cases was obtained from the Scottish government Web site (https://www.gov.scot/coronavirus-covid-19/) and the West of Scotland Specialist Virology Centre. The number of COVID-19–associated deaths was obtained from the National Records of Scotland Web site (https://www.nrscotland.gov.uk/covid19stats).

### Statistical Analysis

Multivariable logistic regression models were used to investigate associations between neutralization at a 1:50 dilution and corrected optical density values, care type, age group, and sex in ELISA positive samples (n = 216). Separate models were run for samples positive to S1 and RBD (Supplementary Tables 2 and 3). Univariate analyses comparing the mean corrected optical density, or percentage neutralization, between ELISA-positive samples from primary and secondary care types were undertaken using Mann-Whitney *U* tests. To determine a sample size for estimating the prevalence of partial postal code districts, we used a simple calculation, assuming a random sample from a large population. An assumed prevalence (*p*) of 10%, and a confidence of 95%, substituted into the equation n* = *1.96^*2*^*p*(1 − *p*)/*d*^2^ (where *d* = precision = 0.05), resulted in a sample size of 138. Statistical analyses and data visualization were undertaken using R software [12], version 3.6.1. Models were run using lme4 package [13].

### Bayesian State-Space Model

A state-space model was developed to estimate the weekly probability of infection of the patient population and to evaluate the impact of the different demographic factors affecting the probability of an individual being seropositive for SARS-CoV-2. The model followed methods published elsewhere [14] and comprised 2 coupled parts: a population-level process and an observation or individual-level process. The population process captured the weekly exposure dynamics through a linear predictor comprising a temporal trend and autocovariates (ie, first- and second-order autoregressive components capable of reconstructing potential exposure cycles). This results in a weekly probability of infection that reflects the average chance of being infected in a given week after adjustment for individual covariates in the observation process.

The observation process confronted the population probabilities by using individual-level data (ie, binary observed serological data from each patient) in a Bernoulli trial that adjusted seropositivity according to the sensitivity and specificity of the test and estimated an individual’s probability of infection based on the population-level dynamics but also through a series of individual covariates such as sex, age, care type and week of sample collection. We noted that since further adjustment for population size resulted in differences of approximately 0.1% in group-based seroprevalence estimates, for simplicity this was omitted from the final state-space model. We ran the model in JAGS for 100 000 iterations and 50 000–iteration burn-in to achieve full convergence. Priors and the model code are provided in the Supplementary Material.

### Infection Fatality Ratio

An infection fatality rate was calculated for each age group by estimating the fraction of SARS-CoV-2–confirmed deaths relative to the number of people exposed. The latter variable was approximated using the adjusted seroprevalence, multiplied by the corresponding group population size (455 739, 310 813, 106 435, and 80 745 for the 18–44-, 45–64-, 65–74-, and ≥75-year age groups, respectively). Mid-2019 population estimates were obtained from the National Records of Scotland (https://www.nrscotland.gov.uk).

## RESULTS

**Figure 1.**
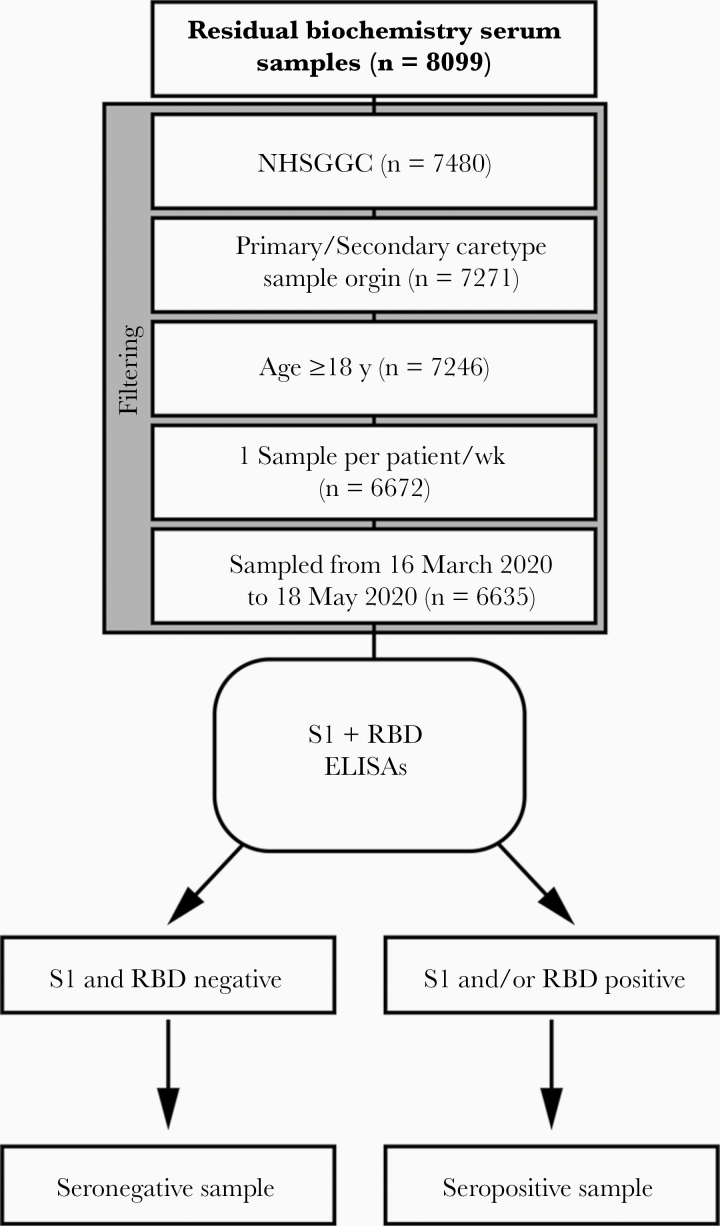
Diagram summarizing the flow of samples used in this study. Abbreviations: ELISAs, enzyme-linked immunosorbent assays; NHSGGC, NHS Greater Glasgow and Clyde; RBD, receptor-binding domain; S1, spike glycoprotein.

A total of 7480 residual biochemistry serum samples from patients living in NHSGGC were tested for the presence of IgG antibodies against the S1 subunit of the SARS-CoV-2 spike protein and its RBD using 2 ELISA assays [9]. Of these, 6635 met the inclusion criteria and were used for further analysis. Samples spanned a 10-week period, starting on 16 March 2020 and covered all NHSGGC districts and all age groups, except for children and young adults <18 years of age for whom insufficient samples were available (Figure 1 describes the sample inclusion criteria and sample sizes). The underrepresentation of samples from pediatric patients reflected the reduction in general practitioner appointments, the prioritization of suspected COVID-19 cases during this period, parents’ avoidance of attending medical facilities to protect children from the virus, and likely reduced risk of non–COVID-19 infections and injuries (the most common reason for emergency attendances in children) owing to physical distancing as well as the lower incidence of clinical signs in children [15, 16].

**Figure 2.**
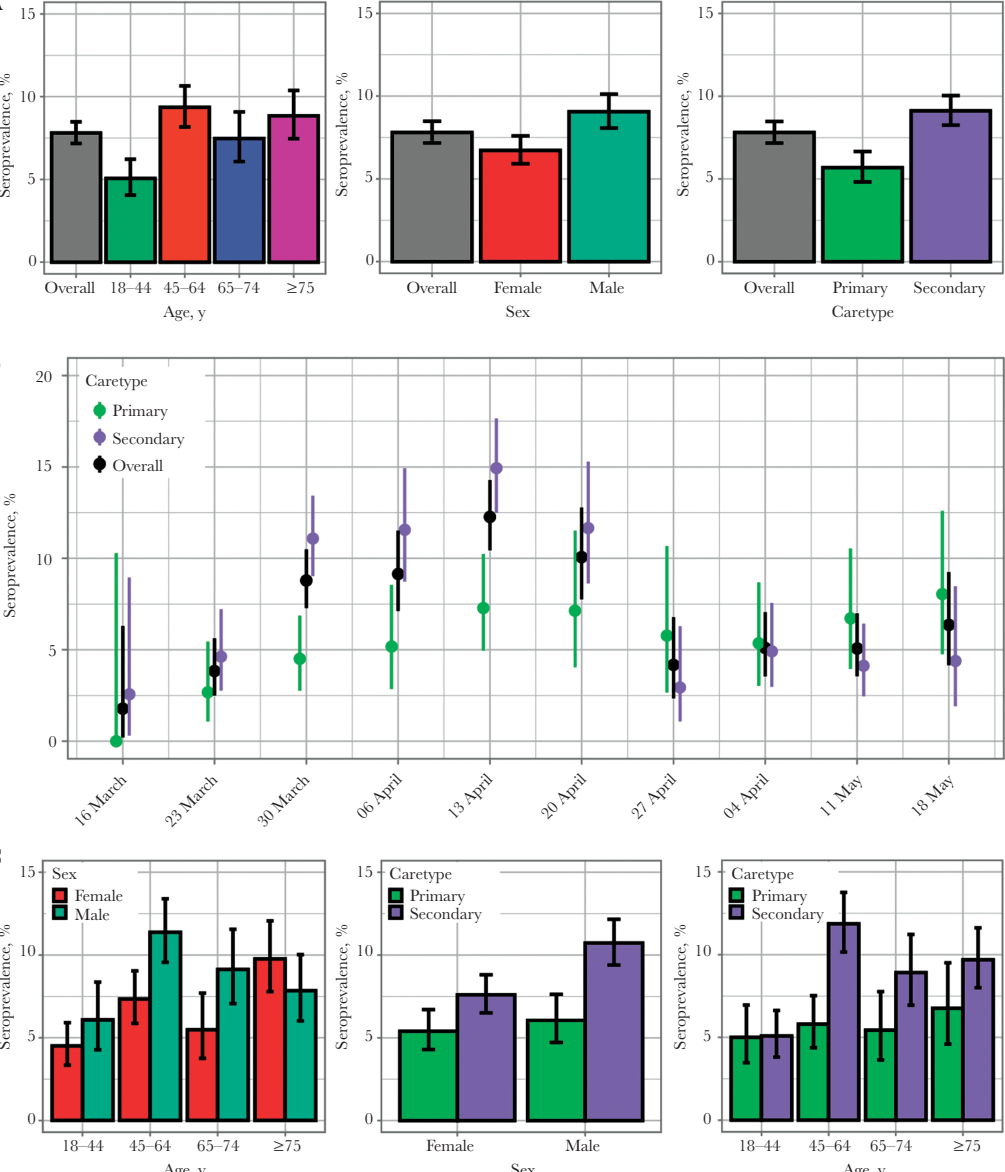
Unadjusted severe acute respiratory syndrome coronavirus 2 (SARS-CoV-2) seroprevalence in NHS Greater Glasgow and Clyde, Scotland, United Kingdom, patient population. *A, B,* Seroprevalence estimates and 95% confidence intervals are shown across age groups, sex. and healthcare setting (*A*), or date of sampling (*B*). *C,* Seroprevalence estimates and 95% confidence intervals investigated in sequential combinations of age group, sex, and healthcare setting.

The overall unadjusted seroprevalence in our patient population was 7.81% (95% CI, 7.17%–8.48%) (Figure 2A). Seroprevalence was higher in 45–64-year-olds, in male patients, and in patients attending secondary care services (Figure 2A). A steady increase in seroprevalence was observed from the week beginning 16 March up to the week beginning 13 April in both primary and secondary care settings. However, while seroprevalence in the secondary care subpopulation was higher, and started to decrease from the week beginning 13 April, seroprevalence in primary care remained at a similar level after the week beginning 13 April to the end of our study period (Figure 2B). For some age groups (45–64 and 64–74 years) seroprevalence was higher in men (Figure 2C), perhaps driven by a sex bias in SARS-CoV-2–associated hospitalization [17], since men admitted to secondary care services had a higher seroprevalence (10.73%; 95% CI, 9.40%–12.17%) than women (7.60%; 6.51%–8.81%) (Figure 2C). This difference between sexes was not observed among primary care patients (6.06% [95% CI, 4.73%–7.63%] for men and 5.40% [4.29%–6.71%] for women) Figure 2C).

**Figure 3.**
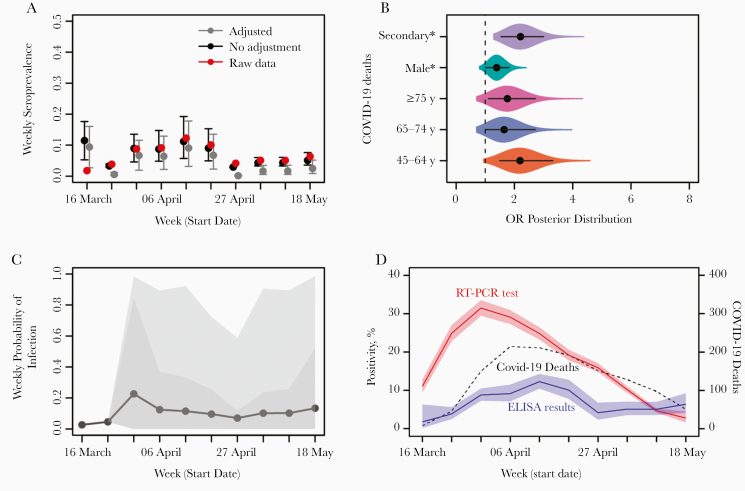
Posterior estimates obtained from the bayesian state-space model. *A,* Model fit (observed data in red vs estimated unadjusted seroprevalence in black) and estimated adjusted seroprevalence (*gray*). *B,* Odds ratios (ORs) of the effect sizes of age, sex and healthcare setting on the probability of a patient being seropositive for severe acute respiratory syndrome coronavirus 2 (SARS-CoV-2) antibodies (95% confidence interval [CI] lines within violin). *C,* Estimated mean weekly probability of infection of the studied population, and associated 75% and 95% CIs. *D,* Unadjusted SARS-CoV-2 seroprevalence (blue), reverse-transcription polymerase chain reaction (RT-PCR)–confirmed coronavirus disease 2019 (COVID-19) cases (red), and COVID-19–related deaths (black) are shown. Abbreviation: ELISA, enzyme-linked immunosorbent assay.

Patient seroprevalence was also calculated in a subset of districts (20 of 61) in which sample numbers provided sufficient power to estimate prevalence. Estimated seroprevalences ranged from 3.83% (95% CI, 1.67%–7.40%) to 12.94% (8.29%–18.94%) (Supplementary Table 1) suggesting that there may be geographically driven differences in infection risk. However, sample size limitations prevented more detailed analysis. Our bayesian state-space model [14] was used to adjust the crude patient seroprevalence rates for the sensitivity and specificity of the assays and to determine the factors associated with seropositivity in the study population. The model converged well and provided a good fit to the data (Figure 3A and Supplementary Figure 2). Although the test had high sensitivity (95.31%; 95% CI, 90.08%–98.26%) and specificity (97.20%; 94.76%–98.71%), the adjusted overall seroprevalence (5.29%; .13%–15.10%) was approximately half the crude estimates (Figure 3A and Table 1). The analysis indicated that patients receiving secondary care were twice as likely (odds ratio, 2.2; 95% CI, 1.6–3.1) to be seropositive as those in primary care (Figure 3B).

**Table 1.** Observed and Adjusted Seroprevalences in the Different Demographic Groups of the Study Population

Demographic Group	Population Size	Samples, No	Seroprevalence, Mean (95% CI), %		COVID-19– Related Deaths, No.	IFR, %
			Unadjusted	Adjusted		
Sex						
Male	459 189	3092	9.06 (8.07–10.12)	6.49 (.16–17.67)	606	NA
Female	494 556	3543	6.72 (5.92–7.59)	4.23 (.13–13.14)	627	NA
Care type						
Primary	NA	2531	5.69 (4.82–6.66)	2.95 (.10–8.23)	NA	NA
Secondary	NA	4104	9.11 (8.25–10.04)	6.73 (.21–17.44)	NA	NA
Age group, y						
18–44	455 739	1662	5.05 (4.05–6.22)	3.10 (.10–9.05)	8	0.06
45–64	310 813	2202	9.36 (8.17–10.65)	6.67 (.16–17.84)	103	0.50
65–74	106 435	1244	7.48 (6.08–9.08)	5.18 (.15–13.98)	164	2.97
>75	80 758	1527	8.84 (7.46–10.38)	5.78 (.17–14.96)	958	20.52
Overall	953 745	6635	7.81 (7.17–8.48)	5.29 (.13–15.10)	1233	NA

Abbreviations: CI, confidence interval; COVID-19, coronavirus disease 2019; IFR, infection fatality rate; NA, not available.

Male patients were 1.39 (95% CI, 1.1–1.8) times more likely to be seropositive, and individuals belonging to the 45–64-year age group were 2.2 (1.5–3.3) times more likely to be seropositive than those in the 18–44-year age group. However, belonging to the older age groups (≥65 years) did not significantly increase the probability of being seropositive (Figure 3B). Nonetheless, considering the adjusted seroprevalences per age group, and their associated population size and SARS-CoV-2–related deaths, we estimated a higher infection fatality ratio in older age groups (Table 1), consistent with findings from a previous United Kingdom–based study [18]. The probability of infection at the population level (Figure 3C) peaked once during the week beginning 30 March, 2 weeks before the week with highest seroprevalence and coincided with the peak of polymerase chain reaction–confirmed cases (Figure 3D). After this peak, there was a low and constant weekly probability of infection (median 10.2%; 95% CI, 3.1%–20.6%) (Figure 3C), likely reflecting the strict lockdown conditions of the study period. At the end of the study period, before lockdown was eased, we observed a slight increase in the probability of infection (Figure 3C), but further data would be required for confirmation. Together, these results suggest that while levels of infection by SARS-CoV-2 remained broadly constant from the introduction of the virus, they were higher among men, 45–64-year-old patients, and those who attended secondary care.

**Figure 4.**
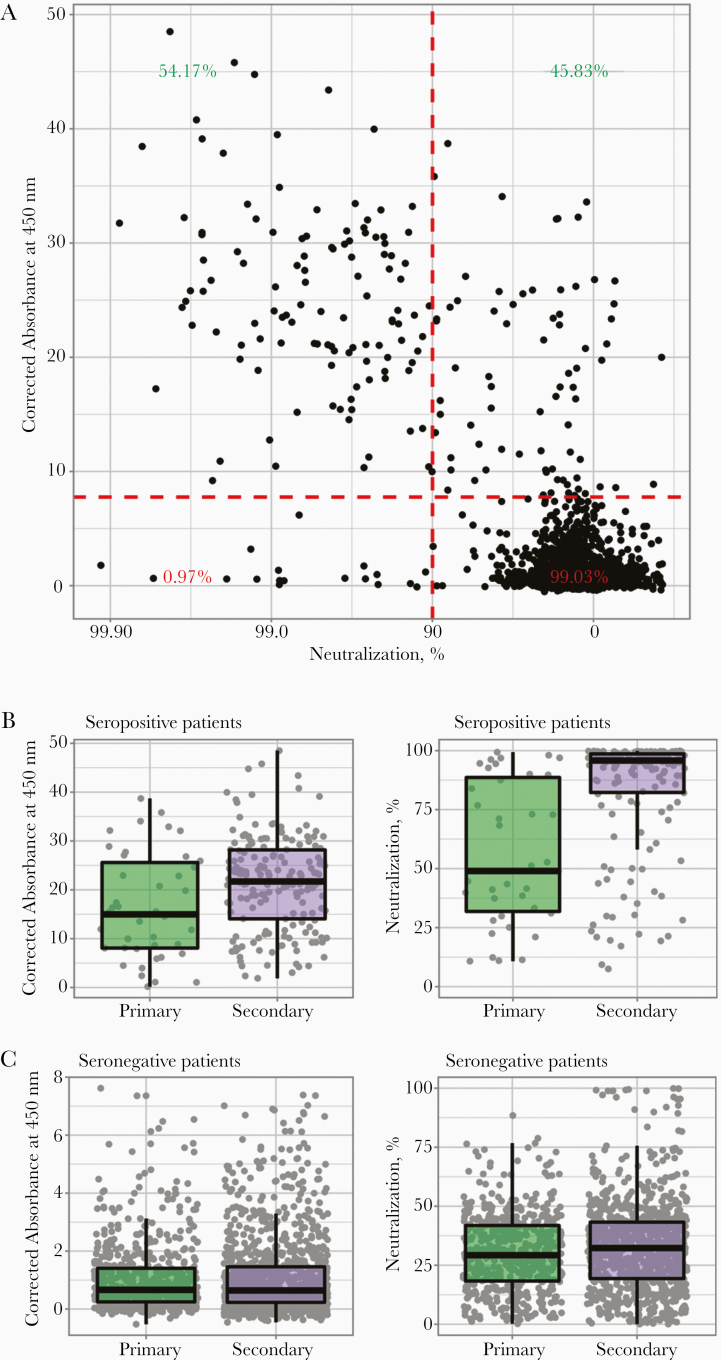
Antibody levels and subsequent virus neutralization activity suggest an association with disease severity. *A,* Correlation between virus neutralization and antibody production is shown as a scatterplot, where every sample is represented by a black dot. Percentages reflect the sample distribution among seropositive patients (*green numbers*) and seronegative patients (*red numbers*), and between low (*right*) and high (*left*) virus neutralization. Enzyme-linked immunosorbent assay corrected-absorbance (left) and virus neutralisation (right) values are shown in patients seropositive *B* or seronegative *C* for acute respiratory syndrome coronavirus 2.

To determine whether exposure might elicit a protective immune response, HIV (SARS-CoV-2) pseudotypes were used to measure levels of neutralizing anti-SARS-CoV-2 antibodies in samples collected between 24 March and 24 April (n = 1974; 10.94% positive by ELISA). A total of 117 (54.17%) ELISA-positive and 17 (0.97%) ELISA-negative samples exhibited high neutralizing activity (Figure 4A). Serum samples were considered to have high neutralizing activity if they reduced infection by >90% at a 1:50 dilution. Overall, our results suggest that approximately half of those individuals who seroconverted elicited a highly neutralizing response. Serum samples with higher absorbance levels in ELISAs exhibited higher levels of virus neutralization (Figure 4A and Supplementary Tables 2 and 3).

In addition, serum samples derived from ELISA-positive patients in secondary care displayed significantly higher mean absorbance values (*P* = .004) (Figure 4B) and mean percentage neutralization than samples from antibody-positive patients in primary care (Figure 4C), implying that disease severity is associated with a stronger and more effective antibody-mediated response. Multivariable logistic regression models confirmed that increasing absorbance values on ELISA were significantly associated with neutralization (odds ratio, 1.15; 95% CI, 1.10–1.21; *P* ≤ .001), and that samples derived from secondary care had significantly higher odds of neutralizing ability than those from primary care (6.77; 2.68–18.75; *P* ≤ .001) (Supplementary Tables 2 and 3).

## Discussion

Serological surveys are key to informing strategies aimed at controlling the spread of disease. Our study showed that SARS-CoV-2 exposure during the first wave of the pandemic remained broadly consistent over time (likely due to lockdown conditions), but heterogeneous among different groups of the Glasgow patient population. After adjustment for test sensitivity and specificity, the overall seroprevalence in the patient population of NHSGGC (5.29%) was similar to reports from community-based cross-sectional studies carried out during an equivalent period in other European cities, such as Geneva [19] and Madrid [20]. However, because our study relied on analyses of residual biochemistry samples from a population of individuals seeking healthcare including—but not exclusively—people who are more likely to be symptomatic with SARS-CoV-2 infection than the general population, generalization beyond the study population requires caution. For example, male patients had a significantly higher risk of being seropositive in our study, although this was not a feature of the previous community-based studies, likely reflecting a sex bias in COVID-19 presentation [21] or differences in social behavior that led to increased exposure [22].

It is important to note that 38% of samples were derived from patients attending primary care, and this proportion remained stable during the studied period. Under normal circumstances, such samples would provide a cost-effective method of obtaining samples for serosurveillance that are broadly representative of the wider community [23]. However, the unprecedented changes to routine healthcare guidelines and health-seeking behavior [16] during the first wave of the pandemic are likely to have altered the structure of this population considerably. Patients in primary care were well enough to be managed in the community and so might be subject to similar exposure conditions as the general population.

At the same time, groups that continued to be seen in primary care for blood sampling, including pregnant women and those with chronic conditions, may have shielded during this period and thus have had lower exposure than the general population. The prevalence in this group may therefore be lower than the expected community prevalence. Conversely, the probability of exposure for individuals from secondary care might be higher than expected in the general population owing to the prioritization of severe COVID-19 cases in hospital settings during this period. In addition, some patients may have been in the early stages of infection and may not have seroconverted at the time of sampling, resulting in an underestimation of seroprevalence in both healthcare settings. Overall, and with the aforementioned caveats, the seroprevalence observed in the primary care subpopulation may be a better representation of the general population than that observed in secondary care.

Neutralization assays provided insight into postexposure antibody-mediated immunity. HIV (SARS-CoV-2) pseudotype-based neutralization assays display a high correlation with live virus-based assays [4]. Although we found a significant correlation between antibody levels and neutralizing activity, we also found, in agreement with other studies [24], that exposure to SARS-CoV-2 resulted in heterogenous responses. As samples from secondary care patients showed both significantly higher antibody levels and odds of neutralization capacity, our results suggest that disease severity may be associated with more effective immune responses. However, antibody levels change over time and our results should be considered within this context. Given the time frame of our study, our results are likely to represent the serological profiles of recent infections. Although our data set did not include clinical information on individual patients, the emphasis on reduction of routine procedures and prioritization of patients with COVID-19 during lockdown makes the secondary care population a suitable proxy for severe SARS-CoV-2 infections. Lower IgG and neutralizing responses in primary care patients could also reflect sampling at earlier points after infection. However, similar results linking disease severity and immune response were reported [25–27].

Neutralizing ability observed in a small number of ELISA-negative serum samples suggests that the presence of epitopes outside the SARS-CoV-2 S1 or RBDs may contribute to the neutralizing response. We note that while there is evidence linking the presence of neutralizing antibodies with protection [28], any inferences between antibody levels and protective immunity should be interpreted with caution. The determinants of a protective immune response to SARS-CoV-2 are unknown and recent studies have suggested that T-cell responses play an important role in SARS-CoV-2 immunity [29]. It has been postulated that between 43% and 70% of the population needs to be immune to SARS-CoV-2 to reach herd immunity [30, 31]. Achieving such levels without vaccination is unlikely in the short term, given that seroprevalence, even among secondary care patients who showed the highest seroprevalence, reached only 6.73% (95% CI, .21%–17.44%). The absence of a strong neutralizing response in a large proportion of seropositive patients raises questions regarding the protective nature of the humoral immune response, highlighting the urgent need for further studies into the duration of neutralizing responses and the relationship between IgG response, neutralizing antibody levels, and protection from reinfection.

Our study provides an insight into the demographic factors that influence SARS-CoV-2 exposure and immunity. The low prevalence observed, combined with the heterogeneity of antibody-mediated neutralizing responses, suggests that in the absence of measures such as vaccination or nonpharmaceutical interventions, future waves of SARS-CoV-2 infection are likely to cause significant burden. Future developments in real-time community serological surveillance systems linked with robust correlations of virus immunity are necessary to design interventions and to prioritize those measures that safeguard public health at a minimal societal and economic cost.

## Supplementary Data

Supplementary materials are available at *The Journal of Infectious Diseases* online. Consisting of data provided by the authors to benefit the reader, the posted materials are not copyedited and are the sole responsibility of the authors, so questions or comments should be addressed to the corresponding author.

jiaa788_suppl_Supplementary_Table_1

jiaa788_suppl_Supplementary_Table_2

jiaa788_suppl_Supplementary_Table_3

jiaa788_suppl_Supplementary_Figure_1

jiaa788_suppl_Supplementary_Figure_2

## Notes

***Acknowledgments.*** We thank Clare Orange, David Murray, Christine Willshire, Lisa Jarvis, and Giada Mattiuzzo for providing the serum samples required to carry out this work. We also thank Matt Turnbull and Suzannah Rihn (MRC–University of Glasgow Centre for Virus Research) for providing the pSCRPSY-hACE2 plasmid.

***Financial support.*** This work was supported by the Medical Research Council of the United Kingdom (grant MC_UU_12014/9), the Wellcome Trust (grant 217221/Z/19/Z), the Biotechnology and Biological Sciences Research Council (grant BB/R004250/1), the Francis Crick Institute (grant FC001061), Georgina Gardner Endowment (grant number 145813-01), John Crawford endowment (grant number 123939-01), and the Department of Health and Social Care (grant BB/R019843/1).

***Potential conflicts of interest.***All authors: No reported conflicts. All authors have submitted the ICMJE Form for Disclosure of Potential Conflicts of Interest. Conflicts that the editors consider relevant to the content of the manuscript have been disclosed.
