# Supplementary material for: Severe Acute Respiratory Syndrome Coronavirus 2 Serosurveillance in a Patient Population Reveals Differences in Virus Exposure and Antibody-Mediated Immunity According to Host Demography and Healthcare Setting
Source: J Infect Dis. 2020 Dec 26;223(6):971–80. doi: 10.1093/infdis/jiaa788 (PMC7798933; doi:10.1093/infdis/jiaa788)
Supplement: jiaa788_suppl_Supplementary_Table_1 [file jiaa788_suppl_supplementary_table_1.docx]

| GGCHB Districts | Number of samples | Unadjusted seroprevalence (95% CI) |
| --- | --- | --- |
| G11 | 208 | 3.85 (1.67-7.44) |
| G12 | 197 | 5.58 (2.82-9.77) |
| G13 | 428 | 8.18 (5.76-11.19) |
| G14 | 209 | 3.83 (1.67-7.40) |
| G15 | 175 | 8.00 (4.44-13.06) |
| G41 | 301 | 6.98 (4.37-10.47) |
| G42 | 283 | 12.01 (8.47-16.38) |
| G43 | 201 | 8.96 (5.39-13.78) |
| G44 | 300 | 7.33 (4.65-10.89) |
| G45 | 170 | 12.94 (8.29-18.94) |
| G46 | 307 | 8.47 (5.61-12.16) |
| G51 | 293 | 8.53 (5.60-12.34) |
| G52 | 376 | 6.91 (4.57-9.97) |
| G53 | 394 | 9.14 (6.48-12.43) |
| G61 | 262 | 5.34 (2.95-8.80) |
| G62 | 150 | 5.33 (2.33-10.24) |
| G73 | 273 | 10.62 (7.23-14.90) |
| G76 | 203 | 6.90 (3.82-11.30) |
| G77 | 262 | 6.11 (3.53-9.73) |
| G81 | 452 | 7.96 (5.64-10.86) |

**Supplementary Table 1:** Unadjusted seroprevalences and 95% CI in partial postcode districts of the NHS Greater Glasgow and Clyde, Scotland, UK, study population with >138 samples.
