# Supplementary material for: Severe Acute Respiratory Syndrome Coronavirus 2 Serosurveillance in a Patient Population Reveals Differences in Virus Exposure and Antibody-Mediated Immunity According to Host Demography and Healthcare Setting
Source: J Infect Dis. 2020 Dec 26;223(6):971–80. doi: 10.1093/infdis/jiaa788 (PMC7798933; doi:10.1093/infdis/jiaa788)
Supplement: jiaa788_suppl_Supplementary_Table_2 [file jiaa788_suppl_supplementary_table_2.docx]

|  | Odds ratio (OR) | 95% confidence intervals | | p value |
| --- | --- | --- | --- | --- |
| S1 corrected absorbance | 1.15 | 1.10 | 1.21 | < 0.001 |
| Primary care | Reference |  |  |  |
| Secondary care | 6.77 | 2.68 | 18.75 | <0.001 |
| Female | Reference |  |  |  |
| Male | 1.18 | 0.59 | 2.34 | 0.642 |
| 18-44 years | Reference |  |  |  |
| 45-64 years | 1.22 | 0.46 | 3.20 | 0.689 |
| 65-74 years | 3.10 | 0.98 | 10.34 | 0.059 |
| 75+ years | 0.95 | 0.32 | 2.78 | 0.919 |

**Supplementary Table 2:** Estimated odds ratios, 95% confidence intervals and p values for explanatory variables, including corrected absorbance values against S1 antigen, in a logistic regression model for neutralising activity.
