# Supplementary material for: Severe Acute Respiratory Syndrome Coronavirus 2 Serosurveillance in a Patient Population Reveals Differences in Virus Exposure and Antibody-Mediated Immunity According to Host Demography and Healthcare Setting
Source: J Infect Dis. 2020 Dec 26;223(6):971–80. doi: 10.1093/infdis/jiaa788 (PMC7798933; doi:10.1093/infdis/jiaa788)
Supplement: jiaa788_suppl_Supplementary_Table_3 [file jiaa788_suppl_supplementary_table_3.docx]

|  | Odds ratio (OR) | 95% confidence intervals | | p value |
| --- | --- | --- | --- | --- |
| RBD corrected absorbance | 1.16 | 1.12 | 1.21 | <0.001 |
| Primary care | Reference |  |  |  |
| Secondary care | 5.32 | 2.08 | 14.93 | 0.001 |
| Female | Reference |  |  |  |
| Male | 1.12 | 0.52 | 2.40 | 0.766 |
| 18-44 years | Reference |  |  |  |
| 45-64 years | 1.40 | 0.50 | 3.89 | 0.520 |
| 65-74 years | 2.77 | 0.82 | 9.79 | 0.106 |
| 75+ years | 1.39 | 0.44 | 4.41 | 0.576 |

**Supplementary Table 3:** Estimated odds ratios, 95% confidence intervals and p values for explanatory variables, including corrected absorbance values against RBD antigen, in a logistic regression model for neutralising activity.
