# Supplementary material for: Severe Acute Respiratory Syndrome Coronavirus 2 Serosurveillance in a Patient Population Reveals Differences in Virus Exposure and Antibody-Mediated Immunity According to Host Demography and Healthcare Setting
Source: J Infect Dis. 2020 Dec 26;223(6):971–80. doi: 10.1093/infdis/jiaa788 (PMC7798933; doi:10.1093/infdis/jiaa788)
Supplement: jiaa788_suppl_Supplementary_Figure_1 [file jiaa788_suppl_supplementary_figure_1.pdf]

**Supplementary Figure 1**

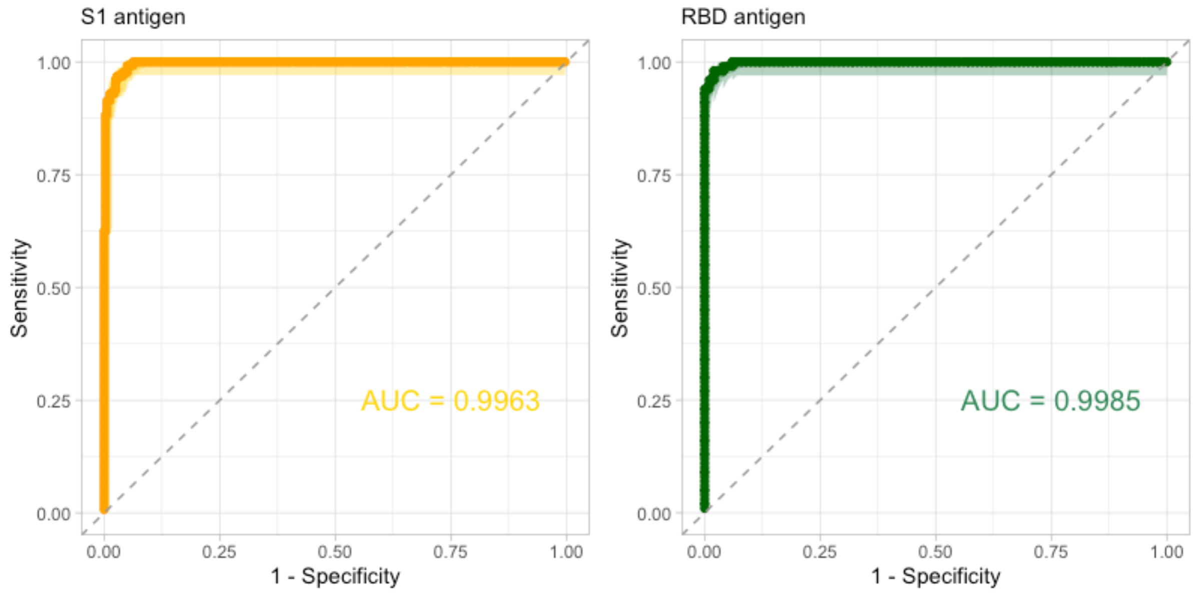


**Supplementary Figure 1**: Receiver operating characteristic (ROC) curves showing the true positive rate (sensitivity) and the false positive rate (1-specificity) of alternative cut-off values for S1 ELISA (left panel), and RBD (receptor binding domain) ELISA (right panel). Data for ROC analyses consisted of corrected-absorbance values for 320 pre-COVID19 negative, and 128 confirmed COVID-19 positive controls. Shading shows 95% confidence intervals.
