# Supplementary material for: Severe Acute Respiratory Syndrome Coronavirus 2 Serosurveillance in a Patient Population Reveals Differences in Virus Exposure and Antibody-Mediated Immunity According to Host Demography and Healthcare Setting
Source: J Infect Dis. 2020 Dec 26;223(6):971–80. doi: 10.1093/infdis/jiaa788 (PMC7798933; doi:10.1093/infdis/jiaa788)
Supplement: jiaa788_suppl_Supplementary_Figure_2 [file jiaa788_suppl_supplementary_figure_2.pdf]

Supplementary Figure 2

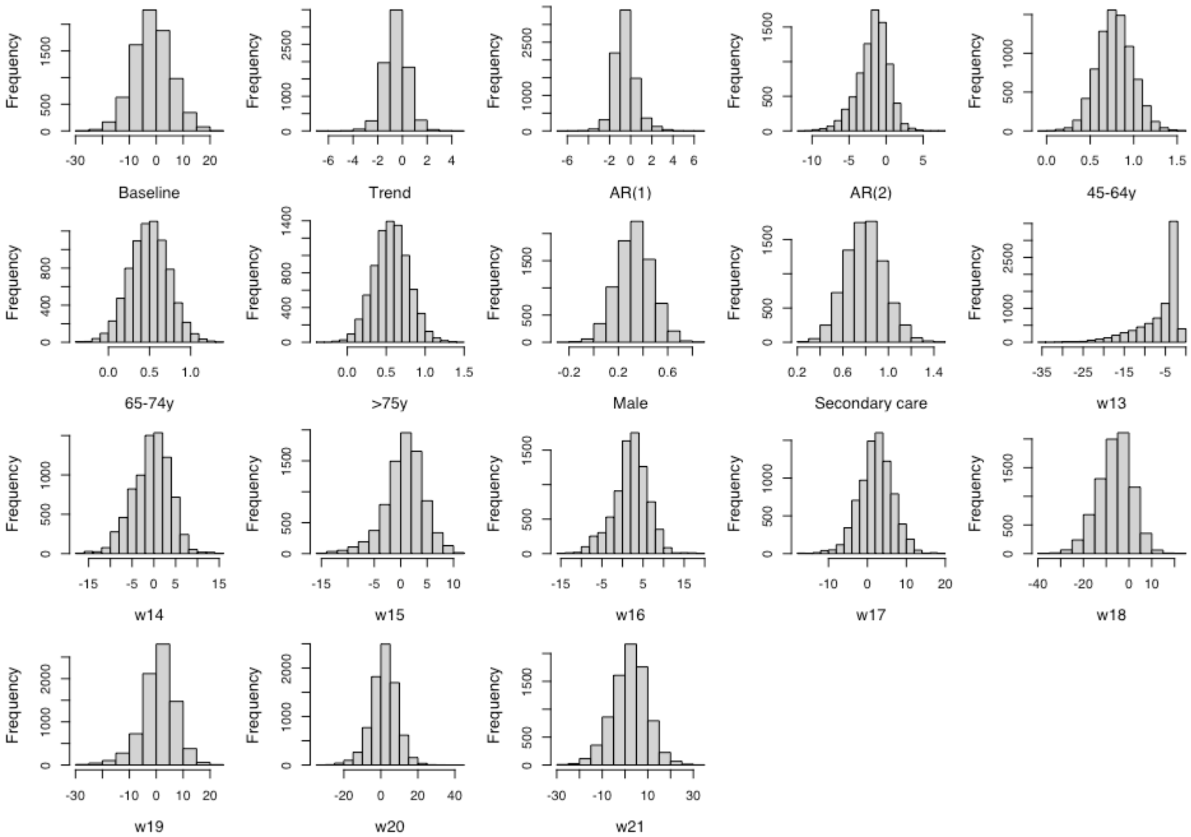

Supplementary Figure 2. State-space model posterior distribution for the key parameters.
